# Changes of free radicals and digestive enzymes in saliva in cases with deficiency in *spleen-yin* syndrome

**DOI:** 10.1016/S1674-8301(10)60035-8

**Published:** 2010-05

**Authors:** Muxin Wei, Yanmin Wu, Dezheng Chen, Yuchun Gu

**Affiliations:** Department of Traditional Chinese Medicine, the First Affiliated Hospital with Nanjing Medical University, Nanjing 210029, Jingsu Province, China

**Keywords:** deficiency in *spleen*-*yin*, deficiency in *kidney*-*yin*, digestive enzymes in saliva, amylase, salivary lysozyme, salivary flow rate, free radicals

## Abstract

**Objective:**

To explore the nature of deficiency in *spleen-yin* syndrome, which could provide scientific theoretical support and practical guidance for clinical Traditional Chinese Medicine (TCM) syndrome differentiation based on biology, and had a strong clinical significance.

**Methods:**

Serum Cu and Zn were detected by atomic absorption spectrophotometer, serum vitamin E by high performance liquid chromatography, serum vitamin C by 2,4-Dinitrophenylhydrazine Colorimetry, total superoxide dismutase (SOD) and Cu and Zn-SOD by the xanthine oxidase method, and malondialdehyde (MDA) by the 2-thiobarbituric acid method (TBA). Total antioxidant capacity was detected by a colorimetry kit. Amylase Activity was detected by an automatic biochemical analyzer. Lysozyme was detected by lysozyme detection plate, the diameter of bacteriolysis circle was measured and the corresponding content of lysozyme was obtained from a table of standard curve values.

**Results:**

No significant difference in total SOD and Cu, Zn-SOD was found between deficiency in *spleen-yin* group and normal group. However, such factors in deficiency in *kidney-yin* group were significantly lower than the other groups (*P* < 0.05). The MDA content in both deficiency in *spleen*-*yin* group and deficiency in *kidney*-*yin* group were significantly higher than that of normal group (*P* < 0.05), while the total antioxidant capacity was significantly lower than normal group (*P* < 0.05). The vitamin E content in deficiency in *kidney*-*yin* group was significantly lower than that in the other two groups (*P* < 0.05). No significant difference in the contents of vitamin C, Cu and Zn were observed in these groups. The Zn/Cu level in deficiency in *kidney*-*yin* group and the vitamin E level in deficiency in *spleen*-*yin* group decreased, but with no significant difference. Amylase activity in unit time in cases with deficiency in *spleen*-*yin* was lower than and had significant differences with that in normal cases, and higher than that in cases with deficiency in *kidney*-*yin*. The sectional velocity of saliva and the ratio of lysozyme in normal case group were significantly higher than other two groups, while deficiency in the *spleen*-*yin* group was significantly higher than the deficiency in *kidney*-*yin* group.

**Conclusion:**

All the results indicated that the objective pathological mechanism between the deficiency in *spleen*-*yin* and deficiency in *kidney*-*yin* was different.

## INTRODUCTION

Deficiency in *spleen*-*yin* syndrome (DSYS) is the most common disease of TCM. Over recent years, people have done a lot of work on DSYS[1]–[6]. To further explore the nature of DSYS, we established the deficiency in *spleen*-*yin* group (DSYG) and the control groups: deficiency in *kidney*-*yin* group (DKYG) and normal control group (NG). Moreover, the nature of DSYS is the lack of *yin points*, which perhaps cause oxygen free radicals damage and changes in digestive enzymes. So we measured some indicators of serum free radicals and digestive enzymes in the saliva.

## MATERIALS AND METHODS

### Subject

#### Deficiency in spleen-yin group

This group was composed of 25 cases, including 15 males and 10 females, whose age ranged from 26 to 70 years with an average age of 45 years. All cases met the diagnostic criteria of DSYG through comprehensive analysis of data obtained by “auscultation, olfaction, inquiry and pulse-taking” four diagnostic methods[7].

#### Deficiency in kidney-yin group

This group was composed of 20 cases, including 11 males and 9 females, whose age ranged from 35 to 75 years with an average age of 50 years. All cases met the diagnostic criteria of DKYG through comprehensive analysis of data obtained by “auscultation, olfaction, inquiry and pulse-taking” four diagnostic methods[7].

#### Normal case group

This group was composed of 30 cases, including 17 males and 13 females, whose age ranged from 35 to 65 years with an average age of 49 years. All cases were chosen from a common healthy population.

There was no statistical significant difference among the three groups in gender and age. Gender was analyzed by chi-square test, χ^2^ = 3.47, *P* > 0.05 while age was analyzed by one-way analysis of variance (ANOVA), *F* = 2.76, *P* > 0.05.

### Sample collection

#### Free radicals

For determination of oxidative damage more objectively from the same baseline, the same batch of samples was collected. For each case, 8 ml venous blood was taken at morning after an overnight fast and the serum was detected after precipitation and separation.

#### Digestive enzymes in saliva

Oral examination was conducted on the subjects to make sure the healthy status of the subjects' oral cavity a few days before sample collection. On the day for sample collection, the subjects were re-examined to ensure that no teeth were hyperemic or bleeding. Sampling was carried out at 8:30-9:30 am after an overnight fast. The subjects rinsed the mouth with warm water 3 times before sample collection and were required to sit upright and not to move heads. Citric acid test paper, 0.5×0.5 cm^2^, was put at the tip of the tongue, and 1.5 ml naturally accumulated saliva under the tongue was collected with a saliva collector. The process was timed. Ten µl saliva was used for detection of amylase and salivary lysozyme.

### Reagents and methods

#### Free radicals

Serum Cu and Zn were detected by atomic absorption spectrophotometer (AA26501, Shimadzu, Japan), serum vitamin E by high performance liquid chromatography (HPLC2996 photodiode array detector, Waters, Ltd, USA), serum vitamin C by 2, 4-Dinitrophenylhydrazine Colorimetry (Du2650 ultraviolet spectrophotometer, Beckman, USA), total superoxide dismutase (SOD) and Cu and Zn-SOD by the xanthine oxidase method, and malondialdehyde (MDA) by the 2-thiobarbituric acid (TBA) method. Total antioxidant capacity (TAC) was detected by a colorimetry kit (Nanjing Jiancheng Bioengineering Institute, Nanjing, China).

#### Digestive enzymes in saliva

Amylase Activity was detected by an automatic biochemical analyzer (Shimadzu, Japan). Lysozyme detection plate was provided by Department of Biochemistry, Nanjing University, Nanjing, China. The diameter of the bacteriolysis circle was measured and the corresponding content of lysozyme was obtained from a table of standard curve values.

### Data analysis and statistics

Experimental data were expressed as mean ± SD. Differences among groups were analyzed by one-way ANOVA, and Scheffe method was used for multiple comparison. The P-value reported was two-sided and a value of *P* < 0.05 was considered statistically significant. All analyses were performed using the SPSS software (Version 11.0, SPSS Inc., USA).

## RESULTS

### Free radicals

**Fig 1:**
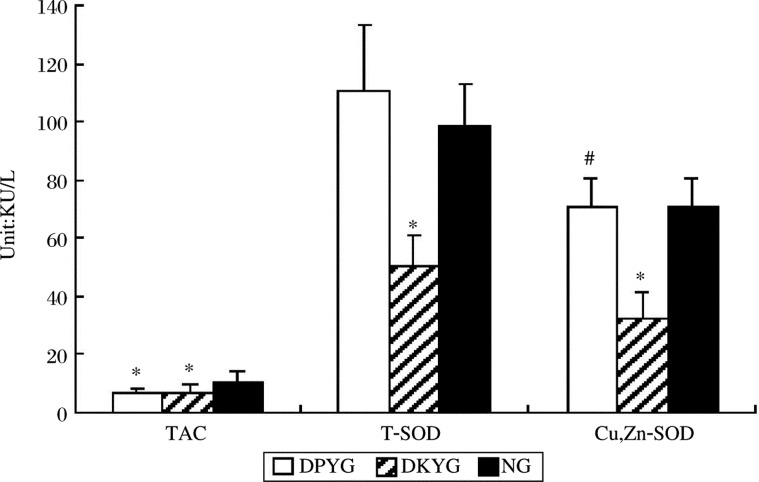
The serum contents of TAC, T-SOD, Cu,Zn-SOD in each group. *Compared with NG, *P* < 0.05; ^#^Compared with DKYG, *P* < 0.05.

**Fig 2:**
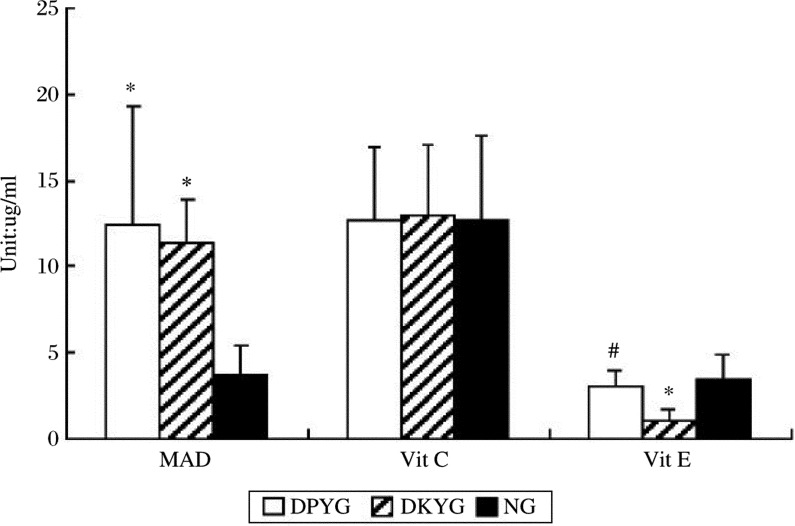
The serum contents of Vit C, Vit E, MAD in each group. *Compared withNG, *P* < 0.05; ^#^Compared with DKYG, *P* < 0.05.

No significant difference in total SOD and Cu, Zn-SOD was found between DSYG and NG. However, such factors in DKYG were significantly lower than those of the other groups (*P* < 0.05) (***Fig 1***). The MDA content in both DSYG and DKYG was significantly higher than that of NG (*P* < 0.05), while TAC (***Fig 1***) was significantly lower than NG (*P* < 0.05) (***Fig 2***). The vitamin E content in DKYG was significantly lower than that in the other two groups (*P* < 0.05) (***Fig 2***). No significant difference in the contents of vitamin C, Cu and Zn were observed among these groups. The Zn/Cu level in DKYG and the vitamin E level in DSYG were decreased, but showed no significant difference.

### Digestive enzymes in saliva

The amylase activity in unit time (AAUT) in cases with DSYG was lower than and had significant differences with that in NG, and higher than that in cases with DKYG (***Table 1***).

The salivary lysozyme content (lz), flow rates (V) and salivary lysozyme content in unit time (Lz/t) of subjects in the three groups are shown in ***Table 1***:

Scheffe one-way ANOVA method was adopted to make comparisons of two groups, and the results showed: ① lz: there were no significant differences among the three groups; ② V: the index was higher in DSYG than that in DKYG with significant differences (*P* < 0.05); it was higher in NG than DSYG and DKYG with significant differences (*P* < 0.01); ③ Lz/t: the index was higher in NG than DSYG and DKYG with significant differences (*P* < 0.05); the index was higher in DSYG than that in DKYG without significant differences (*P* > 0.05);

**Table 1:** The result of amylase activity per unit time, Lz, V, Lz/t in each group, (±SD)

	DSYG (*n* = 25)	DKYG (*n* = 20)	NG (*n* = 30)
AAUT (IU/L)	2134.13 ± 343.51*^#^	951.62 ± 383.17*	3501.63 ± 1099.63
Lz (mg/l)	0190.80 ± 136.86	228.43 ± 97.15	0119.43 ± 126.91
V (ml/min)	0000.27 ± 0.16*^#^	000.13 ± 0.05*	0000.49 ± 0.14
Lz/t (mg/L/min)	0045.84 ± 7.21*	028.11 ± 4.40*	0084.04 ± 19.45

*Compared with NG, *P* < 0.05; ^#^Compared with DKYG, *P* < 0.05.

## DISCUSSIONS

### Free radicals

DSYS is the most common disease of TCM. Over recent years, people have done a lot of work on DSYS. To further explore the nature of DSYS, we did this research.

Free radicals are atoms or groups of atoms with an odd (unpaired) number of electrons, which are produced by metabolism in normal cells[8]. Active free radials in the body include superoxide anion radicals (O^2−^), hydroxyl free radicals (-OH), hydrogen peroxide (H_2_O_2_) and singlet oxygen (-O_2_). Imbalance in the generation and elimination of free radicals will affect and damage the body severely[9]–[11]. Free radicals attack polyunsaturated fatty acids, which are imbedded in the plasma membrane, the decomposition product of which is MDA. MDA has greater toxicity and can affect normal cell function by interacting with phospholipid protein and depositing inside the cells[12],[13]. The accumulation of free radicals can result in development of many diseases[14]–[18].

Tissues and cells have established a set of antioxidant defense systems against free radical damage after long-term evolution, including the antioxidant enzyme system such as macromolecule scavenger and the antioxidant system. Among them, SOD can capture O^2−^ specifically and promptly, then convert it into H_2_O_2_[19],[20]. SOD can be divided into Cu, Zn-SOD and Mn-SOD according to the concentration of microelements such as Cu, Zn and Mn. Meanwhile, natural antioxidants in our body such as vitamin E, vitamin C, coenzyme Q and hydroxyl compounds can capture and deoxidize O^2−^[21]–[23]. SOD and natural compounds complement each other to keep the body from free radical damage[24],[25].

The results showed that MDA increased significantly in both DSYG and DKYG, indicating that these syndromes both resulted in free radical damage with decreased antioxidant capacity and inadequate clearance of lipid peroxides. MDA deposition destroyed the cell submicroscopic structure, which could be the same pathophysiological basis in the two syndromes. However, their ways leading to free radical damage were different. In DSYG, no significant changes in the contents of vitamin E, vitamin C, total SOD, Cu-SOD, Zn-SOD and Zn/Cu were found, which indicated the function of Cu and Zn-SOD. Therefore, we speculated that the free radical damage was caused by abnormality of the peroxidase system, and was limited at the cellular function level since the body's ability to prevent the free radicals damage the peroxidase system. Antioxidants vitamin E decreased significantly in DKYG, indicating that vitamin E as hydrogen donor was not enough to supply hydrogen to deoxidize free radicals. On one hand, other antioxidants and antioxidant enzymes were consumed. On the other hand, it attenuated the vitamin E function. In addition, total SOD, Cu-SOD and Zn-SOD were all decreased significantly, resulting in aging of cases with DKYS and significant organ dysfunction with the damage of submicroscopic structures, insufficient energy from the mitochondria system, protein degeneration, inhibition of DNA and RNA transcription and other pathological changes due to the increase of free radicals. The Zn/Cu decreased, and the imbalanced ratio of Cu and Zn could inhibit SOD activity, causing whole body imbalance, and immune system dysfunction directly.

In conclusion, cases with DSYS and DKYS all had oxygen free radical damage to some extent, but the mechanism of production and elimination was different. DSYS was mainly caused by peroxidase abnormality. However, DKYS was caused by antioxidant enzyme system changes which resulted in extensive oxidative damage, including SOD activity decrease, insufficient antioxidant and imbalance of Zn/Cu. The DSYS and DKYS had different pathological changes. As we observe the number of cases not enough, further in-depth research in this area is needed.

### Digestive enzymes in saliva

It is generally recognized that the point of *yin* is equivalent to the vaginal fluid, endocrine liquid and exocrine fluid; what's more, the spleen has the closest relation to the digestive system. As a result, the study of digestive enzymes will help resolve the nature of DSYS.

The saliva contains a variety of substances: amylase, salivary lysozyme[26], secretory IgA and epidermal growth factors[27], some of which have a protective effect on the digestive tract, and some of which have translational value as diagnostic markers for human diseases[28],[29]. The secretory function of the salivary gland is mainly controlled by the autonomic nervous system, and peptide nerves in regulation is another determining factor[30]. Therefore, the secretion of saliva and saliva amylase are a direct response to the function of the digestive system and an indirect response to the regulation of peptide nerves.

The experimental results showed that the amylase activity among the three groups had no significant differences, while the time and the energy needed for the salivary glands secreted the same amount of saliva were completely different. From the perspective of the saliva and the effects of amylase, transit time for food in the mouth was fixed, but the amount of saliva secretion in the same time was greatly different: The amount of saliva in the NG was almost double that of the DSYG, while the amount of saliva in the DKYG was only half of that in the DSYG, and so was amylase activity per min. The results indicated that during the time of food transit in the oral cavity, the overall effectiveness of saliva and amylase in normal cases was best, followed by DSYG, and cases with DKYG was worst.

Reports on human salivary lysozyme content differ greatly from each other, since there are many factors which can influence the saliva. To keep the same detection condition, the following measures were taken: ① careful examination of the status of the oral cavity and eliminating increases in salivary lysozyme content caused by gum bleeding; ② carefully cleaning oral cavity to avoid interference brought by food remains; ③ all cases taking the upright sitting posture and keeping their heads still (for different body posture may lead to different volume of secreted saliva) and adopting the same amount of time for sample collection (for different body posture may lead to different volume of secreted saliva); ④ most cases with deficiency in *kidney*-*yin* have less saliva and their saliva is too sticky to collect during sample collection. Thus, saliva secreted after stimulation was collected and citric acid test paper was taken as the stimulation method with identical square measure and place to collect naturally accumulated saliva under the tongue. During the process of sample collection, the contamination of food remains on the surface of tongue should be avoided when cases spit saliva.

The experimental results showed that when only detecting the level of salivary lysozyme content in saliva, the indexes in the three groups showed no significant differences; but salivary flow rates of NG, DSYG and DKYG respectively decreased in turn and there were significant differences between different groups, which reflects that salivary flow rates decreased with the rising extent of deficiency in *yin*, that was, salivary flow rates decreased in DSYG and decreased much more in DKYG. In unit time, content of salivary lysozyme content in the saliva in DSYG and DKYG was lower than and had significant differences with that in NG, while there were no significant differences between DSYG and DKYG. It was believed that this index can objectively and sensitively reflect the status of non-specific immunity in DSYG and DKYG. The reasons are: ① the main function of salivary lysozyme, as we know, is non-specific immunity, and the effect of immunity depends on the volume and function of salivary lysozyme content. Normal people have more salivary lysozyme content in unit time and correspondingly have better immunity, while people with DSYS and DKYS have lower salivary lysozyme content in unit time and correspondingly have worse immunity response, thus becoming vulnerable and weak. ② It is not suitable to solely take the volume into consideration and reflect the function with mg/L, if time factor was discarded. Because energy and time cost to produce the same quantity of saliva are different in different bodies, the efficacy of enzyme in unit time is also different. ③ Primary observation has been made on the immunity of cases with DSYS. IgG was found to be lower in DSYG than that in NG, which has the same prediction in this study. But further investigations should be made to study the inner relationship between changes in salivary flow and disease essentials.

All in all, it is believed that content of salivary lysozyme in saliva can objectively and sensitively reflect the status of DSYG and DKYG, and salivary lysozyme content in non-specific immunity decreases in DSYG and DKYG.

In a word, the experimental results showed that DSYS and DKYS exhibit different pathological changes, which would give a scientific theoretical support and practical guidance to clinical TCM syndrome differentiation on biology, and had a strong clinical significance. But the experiment also has certain limitations: sample size was not enough, diseases with no clear diagnostic criteria and so on, it is necessary to determine the kinds of cases, and then expand the sample size for further study.
